# Retinal vascular calibers and correlations with biomarkers in bitches with pyometra-induced systemic inflammatory response syndrome

**DOI:** 10.14202/vetworld.2025.1345-1356

**Published:** 2025-05-25

**Authors:** Fábio Dumit Pizzinatto, Alexandre Pinto Ribeiro, Bianca Eidt Rodrigues, Hanna Rodrigues Miranda, Nathalia de Assis Pereira

**Affiliations:** 1 Postgraduate Program in Veterinary Science, Faculty of Veterinary Medicine, Federal University of Mato Grosso, Fernando Correa da Costa Avenue, 2367, Boa Esperança, Cuiabá, 78.060-900, Mato Grosso, Brazil; 2 Faculty of Veterinary Medicine, Federal University of Mato Grosso, Fernando Correa da Costa Avenue, 2367, Boa Esperança, Cuiabá, 78.060-900, Mato Grosso, Brazil

**Keywords:** canine ophthalmology, interleukin-4, pyometra, retinal microcirculation, systemic inflammatory response syndrome, vascular endothelial growth factor

## Abstract

**Background and Aim:**

Pyometra is a common uterine disease in intact bitches, frequently progressing to systemic inflammatory response syndrome (SIRS). While retinal vascular alterations have been observed in human SIRS cases, similar evaluations are lacking in veterinary medicine. This study aimed to evaluate retinal vascular calibers in bitches with pyometra-induced SIRS (P-SIRS) and explore correlations between retinal measurements and hematological, biochemical, vascular endothelial growth factor (VEGF), and interleukin-4 (IL-4) levels.

**Materials and Methods:**

A prospective observational study was conducted on 40 bitches diagnosed with P-SIRS and 30 clinically healthy controls. Retinal images were captured at admission using a smartphone coupled with a Volk iNView® (Volk®, Mentor, OH, USA) device. Retinal arteriolar and venular calibers within 0.5–1 disk diameter from the optic disk were measured using ImageJ software (https://imagej.net/ij/index.html). Concurrent hematology, serum biochemistry, VEGF, and IL-4 levels were analyzed. Statistical comparisons and correlations were assessed using non-parametric tests and Spearman’s correlation.

**Results:**

Retinal arteriolar calibers were significantly narrower (p = 0.0001) and venular calibers significantly wider (p = 0.0068) in P-SIRS patients compared to controls. Serum VEGF and IL-4 concentrations were markedly elevated in the P-SIRS group (p < 0.05). Retinal venular calibers positively correlated with band neutrophils (p = 0.02), monocytes (p = 0.04), and negatively with albumin (p = 0.008). Retinal arteriolar calibers negatively correlated with segmented neutrophils (p = 0.04) and VEGF (p = 0.0003). No ophthalmoscopically visible retinal lesions were detected.

**Conclusion:**

Bitches with pyometra-induced SIRS exhibited significant retinal microvascular alterations characterized by arteriolar narrowing and venular dilation. These vascular changes correlated with systemic inflammatory markers and VEGF levels, suggesting that retinal vascular assessment may serve as a non-invasive biomarker for systemic inflammation in veterinary patients. Despite microvascular changes, no clinically visible retinal lesions were observed, warranting further longitudinal studies to elucidate their prognostic significance.

## INTRODUCTION

Pyometra is a prevalent uterine disorder in middle-aged to elderly unspayed female dogs, characterized by a pronounced inflammatory response and the accumulation of mucopurulent exudate within the uterine lumen, typically during the luteal phase [1, 2]. This condition leads to systemic, hematological, and imaging alterations, frequently culminating in moderate-to-severe illness [2, 3]. Irrespective of clinical presentation, approximately 74%–94% of affected bitches exhibit signs consistent with systemic inflammatory response syndrome (SIRS), with reported mortality rates ranging from 10% to 13% [2, 4]. SIRS describes a complex physiological response to infectious or noninfectious insults and is diagnosed in canine patients when at least two of the following criteria are met: Tachycardia or tachypnea, deviations in body temperature (hypothermia or hyperthermia), and abnormal leukocyte counts (leukopenia or leukocytosis), with or without an increase in immature neutrophils [1, 2, 4]. Several studies have explored the roles of different biomarkers in bitches with pyometra-associated SIRS, demonstrating significant alterations in serum interleukin-4 (IL-4), IL-8, keratinocyte-derived chemokine, acute phase proteins, and uterine expression of vascular endothelial growth factor (VEGF) [3–7].

The retina, recognized as one of the most metabolically active tissues, offers a noninvasive and accessible window into systemic microvascular health [8]. Advances in fundus photography and image analysis have enabled precise and objective measurement of retinal vascular calibers [9, 10]. Over the past two decades, human clinical studies by Ikram *et al*. [11] and Invernizzi *et al*. [12] have established associations between retinal vascular calibers and various systemic diseases, including diabetic retinopathy, hypertension, cardiovascular disease, stroke, dementia, and infectious diseases of both viral and bacterial origins. Microcirculatory disturbances in the retina have also been reported in patients with SIRS and septic shock [12–16]. In one study, 93% of patients exhibited delayed retinal arterial filling and a higher prevalence of fluorescein-leaking microaneurysms and retinal hemorrhages [14]. Another study observed larger retinal arteriolar calibers and reduced vascular density in septic patients compared to healthy controls, although no differences were noted between survivors and non-survivors after 24 h [16].

In veterinary ophthalmology, limited investigations have addressed retinal vascular changes. Previous studies by Cirla *et al*. [17] and Enache *et al*. [18] in pre-hypertensive and hypertensive cats reported conflicting findings regarding arteriolar and venular calibers and angles. In healthy dogs, retinal vascular architecture has been documented using optical coherence tomography angiography (OCTA) and compared with classical fluorescein angiography (FA) [19]. Although OCTA offers higher resolution imaging, it requires expensive equipment and invasive fluorescein administration [19]. In contrast, smartphone-based retinal photography has emerged as a feasible alternative for documenting retinal changes in resource-limited veterinary settings [20, 21]. Comparative studies by Xu *et al*. [9] and Hu *et al*. [10] in humans have demonstrated that smartphone fundus photography yields measurements comparable to those obtained using high-resolution retinal cameras; however, such validation is lacking in canine patients.

In human medicine, investigations into the association between inflammatory biomarkers and retinal microvascular parameters have yielded inconsistent results [22]. VEGF, a critical mediator of endothelial proliferation, angiogenesis, and vascular hyperpermeability, has been found elevated in dogs with sepsis [23–25]. Nevertheless, one study reported no significant differences in VEGF levels between healthy and SIRS-affected dogs [24]. However, one clinical study demonstrated increased uterine VEGF expression in female dogs with pyometra [3]. Elevated systemic IL-4 levels have also been documented in humans with SIRS and sepsis, as well as in bitches with pyometra, highlighting its potential anti-inflammatory role in the pathophysiology of these conditions [5, 26].

Although the relationship between systemic inflammatory disorders and retinal microvascular alterations has been extensively characterized in human medicine, comparable investigations in veterinary patients remain scarce. Specifically, while human studies have demonstrated that retinal vascular caliber changes can serve as noninvasive indicators of systemic inflammation and organ dysfunction, no previous studies have systematically evaluated these parameters in dogs affected by pyometra-induced SIRS (P-SIRS). In addition, while smartphone-based fundus imaging has been validated for human clinical use, its application for retinal vascular assessment in dogs with systemic inflammatory diseases has not been thoroughly explored. Furthermore, the association between retinal vascular measurements and systemic inflammatory biomarkers, including VEGF and IL-4, remains poorly understood in veterinary patients.

Therefore, the present study aimed to (i) assess potential retinal abnormalities and quantitatively evaluate retinal vascular calibers in bitches diagnosed with pyometra-induced SIRS; (ii) compare retinal vascular parameters between affected patients and healthy controls using smartphone-based retinal imaging; and (iii) investigate correlations between retinal vascular measurements and systemic hematological, biochemical, VEGF, and IL-4 profiles. By addressing these objectives, this study seeks to determine whether retinal vascular alterations could serve as non-invasive markers of systemic inflammatory status in canine patients.

## MATERIAL AND METHODS

### Ethical approval and informed consent

This study was conducted in accordance with the Guide for the Care and Use of Animals in Research and Teaching and was approved by the Ethics Committee of the Federal University of Mato Grosso (UFMT) (Protocol No. 23108.098827/2022-66). All examined bitches were privately owned, and written informed consent was obtained from the owners before inclusion. All clinical and ophthalmological examinations adhered to good veterinary practice guidelines and were non-invasive and non-painful.

### Study period and location

This prospective study was conducted from December 2022 and August 2024 and included female dogs admitted to the Veterinary Teaching Hospital of UFMT.

### Patient selection

For each patient, data regarding age, breed, reproductive status, owner consent, and clinical diagnosis were recorded. Physical examinations were performed by general practitioners and residents from the small animal section. All ophthalmic examinations were performed by residents from the ophthalmology section and a certified member of the Brazilian College of Veterinary Ophthalmologists.

The physical examination comprised measurements of heart rate, respiratory rate, blood pressure (petMAP graphic®, Ramsey Medical Inc., USA), and rectal temperature. Ophthalmic evaluation included assessment of menace and dazzle reflexes, direct and consensual pupillary light responses, and Schirmer’s tear test (Schirmer’s Tear Test Strips®, Ophthalmos, São Paulo, Brazil). Intraocular pressure was measured using rebound tonometry (Tonovet®, iCare, Finland), and anterior segment structures were evaluated with slit-lamp biomicroscopy (SL-15®, Kowa, Japan). Following a 2-min acclimation period in a darkened environment, chromatic pupillary light reflex testing was performed using red and blue light stimuli (RetinoGraphics BPI-50®, Connecticut, USA). Pupillary dilation was subsequently achieved with 1% tropicamide (Mydriacyl®, Alcon, Brazil) to facilitate indirect ophthalmoscopy (FOH-5®, Eyetec, São Carlos, Brazil). Any clinical or ophthalmic abnormalities were recorded. Complete blood counts and serum biochemical profiles were also obtained. All retinal imaging for vascular caliber analysis was conducted at the time of admission.

### Criteria for SIRS diagnosis

The diagnosis of SIRS was based on the presence of two or more criteria according to established veterinary standards [2]: Heart rate exceeding 140 beats/min in small breeds or 120 beats/min in large breeds; respiratory rate above 30 breaths/min in small breeds or 20 breaths/min in large breeds; body temperature outside the range of 38°C–39.2°C; and leukocyte counts above 16,000 or below 6,000 × 10^3^/μL, or band neutrophil counts exceeding 0.3 × 10^3^/μL [25, 26].

### Enrollment and exclusion criteria

Bitches presenting with clinical signs of SIRS and ultrasonographic findings suggestive of pyometra – such as uterine enlargement, hypoechoic or anechoic luminal contents, or endometrial cysts – were considered for enrollment. Pyometra diagnosis was confirmed intraoperatively during ovariohysterectomy and by histopathological examination [27]. Exclusion criteria included prior systemic or ophthalmic treatments (e.g., eye drops, fluid therapy, analgesics, anti-inflammatories, corticosteroids, antibiotics, and vitamins), concurrent infectious diseases (distemper, leishmaniosis, ehrlichiosis, and babesiosis), diabetes mellitus, or neoplastic disease [25, 26]. In addition, only dogs with clear ocular media and negative fluorescein dye tests were included in the study. The control group (CG) consisted of bitches without SIRS and without uterine abnormalities on ultrasound, matched to the same ophthalmic criteria. Only female dogs were enrolled to eliminate sex-related bias.

### Assessment of retinal vessel caliber

Retinal imaging was performed at the time of patient admission using a smartphone (iPhone 6S®, Apple Inc., Cupertino, USA) coupled with a Volk iNView® (Volk®, Mentor, OH, USA) device under standardized low-light conditions. Animals were manually restrained, and video recordings were taken without filters and with the camera flash enabled. The best-quality still image, based on vasculature focus and optic disk centering, was selected for analysis. Images were cropped at the retinal circle margins and anonymized using a randomization website (http://www.randomization.com). Two independent observers, blinded to patient groups, analyzed the images using ImageJ software (http://www.rsbweb.nih.gov/ij/). A second evaluation was conducted by the same observers 2 weeks after the initial assessment.

### Calibration and measurement methodology

**Figure 1:**
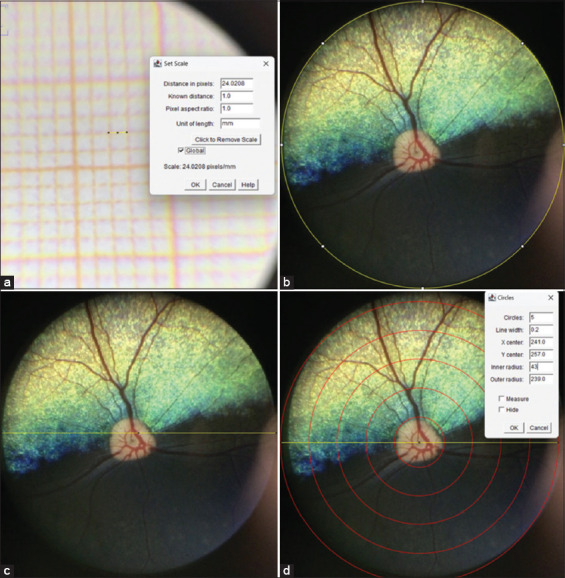
(a) Calibration was performed using millimetric paper, and the field of view was set to 24.0208 pixels, corresponding to 1.0 mm (1,000 µm). (b and c) The center of the retinal image was found using circular and straight tools: (d) The center of the bar was placed at the center of the optic disk; the inner radius corresponding to optic disk diameter was calibrated, which automatically generated the area to be evaluated, located between half and one disk diameter distance from the optic disk.

**Figure 2:**
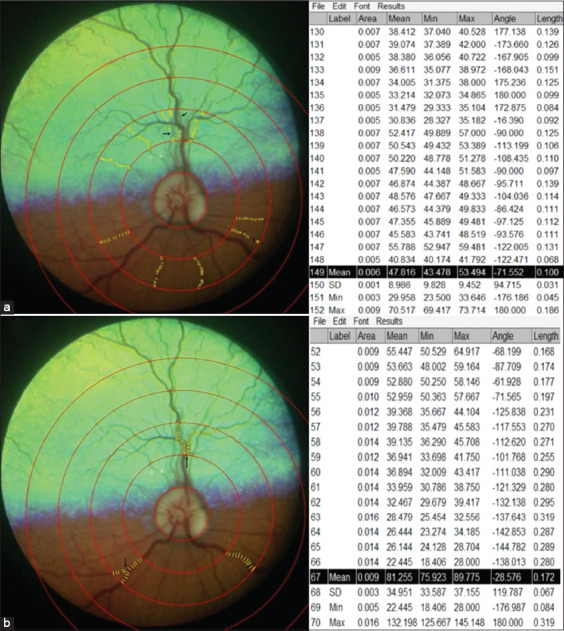
The caliber of all visible (a) arterioles and (b) venules were separately assessed using a straight tool along their respective paths. (a) Due to artifacts or blurring in the images, some vessels (mainly arterioles) were measured at only certain points (arrows). (b) Points located after a single bifurcation were measured, but the arteriolar and venular branches were not (arrows). In this image from a control patient, (a) 148 points were measured from 10 different arterioles and (b) 66 points from three different venules.

Calibration was performed using a millimetric paper image captured under identical settings, setting the width of 1.0 mm (1,000 μm) to 24.0208 pixels (Figure 1a). The retinal image center was determined using circular and straight-line tools (Figures 1b and c). The concentric circles plugin was applied, calibrating the inner radius to the optic disk diameter and automatically generating a measurement region between 0.5 and 1 disk diameter from the optic disk (Figure 1d). The calibers of all visible arterioles and venules within this zone were measured along their paths (Figures 2a and b). All vessels were initially marked collectively but annotated separately for objectivity. Caliber was calculated as the mean value across vessel segments. Branch points were not included unless located after a primary bifurcation. Image artifacts occasionally limited measurements, primarily in arterioles.

### Intra-and interobserver variability

Inter- and intra-observer reliability was assessed using retinal images from 30 randomly selected female dogs (15 from CG and 15 from the P-SIRS group). Both observers (FBP and APR) independently annotated vessel diameters at identical locations, blinded to the clinical status of the animals. Bland-Altman plots and Pearson correlation coefficients were used to assess agreement.

### Quantification of VEGF and IL-4 levels in serum

Serum samples were thawed at room temperature (25^o^C) and diluted 1:5 before analysis. VEGF and IL-4 concentrations were determined using commercial ELISA kits: Canine VEGF (FineTest®, Hubei, China) and Canine IL-4 Sandwich (LS Biosciences®, Massachusetts, USA), respectively, following manufacturers’ instructions. Absorbance was measured at 450 nm, and concentrations were calculated using four-parameter logistic curve fitting (www.myassays.com). Detection limits were 31.25–2,000 pg/mL for both assays. Coefficients of variation were IL-4: Intra-assay <5.5%, inter-assay <8.4%; VEGF: Intra-assay 5.05–5.30%, inter-assay 4.98%–5.27%.

### Statistical analysis

Data from both observers were averaged for statistical analysis using Prism 4.0 (GraphPad Software Inc., California, USA). Normality was assessed with the Shapiro-Wilk test. Differences between CG and P-SIRS groups in clinical, laboratory, and retinal parameters were evaluated using the Mann–Whitney U-test. Spearman’s rank correlation coefficient was used to assess relationships between retinal vessel calibers and laboratory or cytokine findings. Pearson correlation coefficients and Bland-Altman plots were employed to assess inter- and intra-observer variability. Statistical significance was set at p < 0.05. Data are presented as median and range.

## RESULTS

### Study population

Forty bitches met the inclusion criteria and were allocated to the P-SIRS group. In these patients, the diagnosis of P-SIRS was confirmed by post-operative macroscopic evaluation of an enlarged and pus-filled uterus. Thirty bitches without P-SIRS (25 spayed and 5 unspayed), admitted for elective spay surgery (3), umbilical hernia (2), vaccination (3), and ophthalmologic evaluation for mild entropion (13) and mild caruncular trichiasis (9), met the inclusion criteria and were allocated to the CG. The deformities observed in some patients in the CG (umbilical hernia and palpebral and/or adnexal abnormalities) did not affect retinal vascular changes, as none of the patients in this group showed signs of SIRS or ocular inflammation. In the remaining three patients in the CG who were admitted for vaccination, retinal photographs were taken before the administration of the agents.

The IOP of CG [16.0 (11–21.0 mmHg)] and P-SIRS patients [16.0 (9.0–19.0 mmHg)] did not differ (p = 0.28). Mixed breeds and Shih Tzus were overrepresented in both groups, whereas the incidence of Doberman Pinschers was higher in the P-SIRS group (Table 1). The other breeds included in each group are described in Table 1.

**Table 1:** Breeds enrolled in the control and P-SIRS groups.

Breeds	Control	P-SIRS
Mixed breed	12	17
Fox Houd	1	-
Akita	1	-
Shi Tzu	6	6
Lhasa Apso	1	3
Doberman Pincher	2	6
French Bulldog	1	-
Brazilian Fila	1	-
Poodle	1	-
Pit Bull terrier	1	1
Yorkshire terrier	1	1
German Spitz	-	1
Chihuahua		1
Blue Healer	-	1
Labrador Retriever	-	1
Schnauzer Standard	-	1
German Shepperd	1	1
Dachshund	1	-
Total	30	40

P-SIRS=Pyometra-induced systemic inflammatory response syndrome

There were no significant differences in age (p = 0.86), weight (p = 0.09), or mean blood pressure between groups (Table 2). Patients in the P-SIRS group showed significantly higher cardiac rates, total leukocyte and band and segmented neutrophil counts, total protein, urea, and alkaline phosphatase levels (p < 0.05) (Table 2). In addition, patients in this group had significantly lower values of erythrocytes, hemoglobin, hematocrit, and albumin (p < 0.05) (Table 2). No chorioretinal abnormalities were found in patients in either the CG or P-SIRS groups with regard to hypo- or hyperreflectivity, foci of detachment, or alterations in the optic nerve head.

**Table 2:** Values of clinical parameters, blood work, serum biochemical analysis, and cytokine levels in the control and P-SIRS groups.

Variables	Reference	Control	P-SIRS	p-value
Ages (years)	-	7.0 (1.0–14. 0)	7.0 (1.0–14.0)	0.86
Weight (kg)	-	9.45 (2.5–46.0)	6.80 (1.65–29.1)	0.09
Cardiac rate (beats per minute)	120–140	114.0 (88.0–149.0)	128.0 (80.0–200.0)	**0.008**
Respiratory rate (breaths per minute)	20–30	37.0 (28.0-62.0)	40.0 (20.0–150.0)	0.37
Temperature (°C)	38–39.2	38.35 (37.9–39.4)	38.5 (36.70–40.70)	0.79
Mean blood pressure (mmHg)	<140	117 (81.0–127.0)	109 (71.0–126.0)	0.59
Erythrocytes (×10^6^/μL)	5.7–7.4	6.88 (5.59–8.45)	6.04 (2.29–8.33)	**<0.0001**
Hemoglobin (g/dL)	12–18	15.60 (12.70–20.0)	13.15 (5.30–19.90)	**<0.0001**
Hematocrit (%)	37–55	46.0 (37.0–59.0)	37.50 (16.0–61.0)	**<0.0001**
Total Leucocytes (×10^3^/μL)	6–17	9.40 (4.5–17.10)	18.15 (2.10–74.70)	**<0.0001**
Band neutrophils (×10^3^/μL)	0.0–0.3	0.0 (0.0–0.7)	0.30 (0.0–10.0)	**0.0003**
Segmented neutrophils (×10^3^/μL)	3.0–11.5	5.33 (2.57–10.65)	11.16 (0.88–44.82)	**<0.0001**
Monocytes (×10^3^/μL)	0.1–1.0	0.43 (0.0–0.59)	1.14 (0.12–3.85)	**<0.0001**
Platelets (×10^3^/μL)	175–500	318.0 (135.0–462)	258.0 (30.0–774.0)	0.06
Total protein (g/dL)	5.5–8.0	8.20 (6.40–10.0)	8.90 (6.20–12.0)	**0.02**
Albumin (g/dL)	3.2–4.1	3.70 (2.85–4.40)	2.90 (1.50–4.10)	**<0.0001**
Urea (mg/dL)	21–59.9	33.0 (18.0–66.0)	68.0 (19.0-289.0)	**0.03**
Creatinine (mg/dL)	0.5–1.5	1.00 (0.70–1.50)	1.10 (0.50–5.20)	0.46
Alanine aminotransferase (UI/L)	21–102	44.0 (16–139.0)	34.00 (6.00–479.0)	0.054
Alkaline phosphatase (UI/L)	20–156	74.0 (45.0–156.0)	105.0 (46.0–549)	**0.0007**
VEGF (pg/mL)	-	47.11 (13.16–73.11)	129.2 (14.33–459.0)	**0.03**
IL-4 (pg/mL)	-	0.00 (0.0–45.37)	147.7 (101.5–251.4)	**<0.0001**

VEGF=Vascular endothelial growth factor, P-SIRS=Pyometra-induced systemic inflammatory response syndrome, Bold values are statistically significant (p < 0.05).

### Inter- and intra-observer variability

Inter-observer agreement in 30 retinal images (n = 15 controls and n = 15 P-SIRS) showed strong correlations between the first and second annotations for both observers (p < 0.0001) (Table 3). Bland-Altman analysis indicated that both observers tended to underestimate the arteriolar and venular calibers in their second annotations (Table 3).

**Table 3:** Inter-observer agreement (1^st^ annotation vs. 2^nd^ annotation) for retinal arteriole and venule calibers and angles in a sample population (n = 15 controls, n = 15 pyometra-induced systemic inflammatory response syndrome).

	Observer 1	Observer 2
R pearson (95% confidence interval)		
Caliber of arterioles	0.86 (0.7400–0.9360)	0.73 (0.5496–0.8517)
Caliber of venules	0.85 (0.7069–0.9268)	0.91 (0.8336–0.9607)
Bias (standard deviation)		
Caliber of arterioles	−1.30 ± 11.29 μm	−8.00 ± 17.00 μm
Caliber of venules	−10.82 ± 23.82 μm	−2.10 ± 15.84 μm

Annotations between observers 1 and 2 (intra-observer agreement) also showed significant correlations (p < 0.02) (Table 4). Bland-Altman analysis indicated that observer 2 tended to underestimate the arteriole calibers and overestimate the venular calibers (Table 4).

**Table 4:** Intra-observer agreement (observer 1 vs. observer 2 for retinal arteriole and venule calibers and angles in a sample population (n = 15 controls, n = 15 pyometra-induced systemic inflammatory response syndrome).

Retinal vessel calibers	R pearson (95% confidence interval)	Bias (standard deviation)
Caliber of arterioles	0.42 (0.0753–0.6805, p *=* 0.01)	−2.72 ± 21.06 μm
Caliber of venules	0.85 (0.7174–0.9298, p *<* 0.0001)	14.93 ± 23.33 μm

### Values of retinal vessels between groups

**Figure 3:**
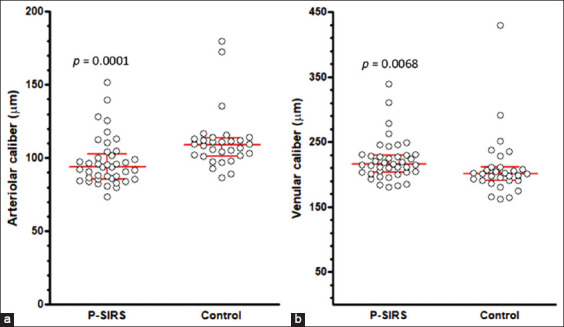
(a and b) Medians, interquartile ranges, and dispersions of vessel calibers in the pyometra-induced systemic inflammatory response syndrome and control groups.

The number of arteriolar and venular points assessed in the CG and P-SIRS groups did not differ significantly (p > 0.05) (Table 5). In the P-SIRS group, the caliber of the retinal arterioles was significantly smaller than that of the CG (p = 0.0001) (Figure 3a) (Table 5). In contrast, the caliber of the retinal venules was significantly larger in the P-SIRS group than in the CG (p = 0.0068) (Figure 3b).

**Table 5:** Number of points assessed and caliber of retinal vessels in the P-SIRS and control Groups.

	P-SIRS	Control	p-value
Points assessed in arterioles	57.88 (17.00–115.5)	59.00 (26.50–149.0)	0.96
Points assessed in venules	40.00 (21.00–66.25)	34.13 (15.25–66.50)	0.15
Arterioles (μm)	93.88 (73.75–151.5)	109.1 (86.50–179.8)	**0.0001**
Venules (μm)	215.8 (181.0–339.3)	201.4 (162.0–429.5)	**0.0068**

P-SIRS=Pyometra-induced systemic inflammatory response syndrome, Bold values are statistically significant (p < 0.05).

### Quantification of VEGF and IL-4 levels in serum

VEGF levels were detected in 9/30 blood samples in the CG and in 12/40 samples in the P-SIRS group. VEGF levels were significantly higher (p = 0.03) in the P-SIRS group (129.2 [14.33–459.0 pg/mL]) than in the CG (47.11 [13.16–73.11 pg/mL])] (Table 2). In the IL-4 analysis, all 25 samples from castrated females in the CG were negative, while 2/5 samples from unspayed females in the CG were positive (37.81 and 45.37 pg/mL). In the P-SIRS group, IL-4 was detected in 10/40 samples and was significantly higher (147.7 [101.5–251.4 pg/mL]) than in the CG (p < 0.0001) (Table 2).

### Correlations among retinal vessel calibers, selected parameters, and cytokines

Retinal venular calibers correlated significantly with band neutrophils (p = 0.02), monocytes (p = 0.04), and albumin (p = 0.008). Retinal arteriolar calibers correlated with segmented neutrophils (p = 0.04) and VEGF (p = 0.0003). Table 6 summarizes the results of correlations between retinal vessels and other parameters.

**Table 6:** Correlations between retinal vessels and selected parameters.

Variables	Retinal arterioles	Retinal venules
Ages	−0.11 (p = 0.33)	−0.06 (=0.57)
Mean arterial pressure	−0.02 (p = 0.84)	−0.06 (p = 0.63)
Platelets	0.12 (p = 0.33)	−0.24 (p = 0.053)
Total Leukocytes	−0.15 (p = 0.02)	0.12 (p = 0.29)
Band neutrophils	−0.05 (p = 0.65)	0.27 (p = **0.02**)
Segmented neutrophils	−0.24 (p = **0.04**)	0.16 (p = 0.17)
Monocytes	−0.10 (p = 0.37)	0.24 (p = **0.04**)
Albumin	0.09 (p = 0.45)	−0.32 (p = **0.008**)
VEGF	−0.68 (p = **0.0003**)	−0.24 (p = 0.25)
IL-4	−0.21 (p = 0.56)	−0.49 (p = 0.15)

Bold values are statistically significant (p < 0.05).

## DISCUSSION

### Retinal vascular measurement and software considerations

The development of semi-automated software platforms enables consistent measurement of various retinal vascular features, such as fractal dimension, branching angles, tortuosity, bifurcation, and vascular length-to-diameter ratio [28, 29]. However, these semi-automated calculations are only available in paid versions of the software, and most platforms require high-resolution retinal images [28, 29]. Despite requiring substantial user input and manual vessel tracing, ImageJ remains freely available and enables the assessment of vessels captured using a smartphone.

In the present study, measurements of the same vessel segment performed by the same annotator at different time points exhibited a good correlation coefficient. The intra-observer correlation coefficient also indicated good repeatability between the two annotators for all parameters, except arteriole caliber, which, although lower, remained statistically significant. This discrepancy may have resulted from difficulties in obtaining a clear visualization of arteriole walls, as previously reported by Enache *et al*. [18].

Changes in vascular calibers are unlikely to have arisen from inter- or intra-observer variability, as the number of points assessed in the arterioles and venules was similar between the groups. The second evaluation was performed after a 2-week interval, with observers blinded to group allocation. Moreover, underestimations and overestimations related to bias are unlikely to have influenced statistical outcomes, as both healthy and diseased retinas were used to establish bias and correlations.

### Smartphone retinal imaging and image quality

Xu *et al*. [9] and Hu *et al*. [10] have demonstrated that retinal vessel measurements obtained from smartphone fundus photography are comparable to those from high-resolution retinal cameras. One study reported that the Volk iNView system offers the largest field of view (75%) and superior image quality compared with the Peek Retina (69%), D-Eye (62%), and iExaminer (61%) systems [30]. Although the manufacturer claims that the Volk iNView system captures high-quality images without pupillary dilation, we encountered difficulties under these conditions.

Consequently, we opted to perform all examinations after pupil dilation, as previous studies by Tsui *et al*. [31] and Frost *et al*. [32] have shown that topical instillation of 1% tropicamide does not affect retinal vessel calibers.

### Selection of measurement area

The VAMPIRE platform was previously utilized by Cirla *et al*. [17] and Enache *et al*. [18] for evaluating healthy and hypertensive cats; however, the algorithms used in these studies are no longer available. In those studies, retinal vessels were assessed at three to four standard measurement points along the vessel paths, equidistant from the optic disk border.

In the present study, this methodology proved impractical, as the canine retina presents multiple scintillant and scattered points, rendering measurements difficult or impossible in certain regions.

Therefore, we selected Zone B – approximately 0.5–1 disk diameter from the optic disk – for vessel evaluation and measured all visible points within this region. This zone offers a balance between proximity to the optic disk, ensuring good vessel visibility, and a distance where vessel calibers have stabilized, better reflecting systemic microcirculatory conditions [10–13, 28, 29].

### Retinal vessel caliber in dogs compared to other species

To date, no studies have assessed the caliber of retinal vessels in dogs. Because previous feline studies reported retinal vessel diameters in pixels, direct comparisons are not feasible [17, 18].

In the present study, retinal arteriole calibers in the CG (109.1 [86.5–179.8 μm]) were smaller than typical human arteriole measurements (145.14 [90.6–172.7 μm]), while venular calibers (201.4 [62.0–429.5 μm]) were comparable to those reported in humans (200.43 [120.4–226.34 μm]) [10–13, 28, 29].

Although similar retinal areas were assessed, vessel caliber calculations employed different algorithms, and human studies included both sexes [10–13,28,29]. As only female dogs were evaluated herein, further research is necessary to determine whether sex influences retinal vessel dimensions.

In addition, the authors acknowledge that the absence of refractive media (cornea, aqueous humor, lens, and vitreous) could introduce bias when calibrating the pixel-to-millimeter conversion. Nevertheless, analyses in humans have shown significant inter-software variability, underscoring the need for validation studies [28,29].

### Microcirculatory dysfunction in P-SIRS and retinal findings

Canine patients with either septic or non-septic SIRS frequently present with microcirculatory dysfunction, characterized by reduced capillary vessel density and impaired red blood cell perfusion across arterioles, venules, and capillaries [24, 25]. In addition, cytokine-induced dysregulation compromises vascular homeostasis, resulting in heterogeneous blood flow distribution, localized tissue hypoxia, and progressive organ dysfunction [24, 25]. In this study, P-SIRS patients exhibited retinal venular dilation, consistent with observations in human SIRS and sepsis cases [11–13, 33].

However, contrary to three human studies [12,13,33], we observed retinal arteriolar constriction rather than dilation. Ikram *et al*. [11] and Simkiene *et al*. [15] reported arteriolar vasodilation in SIRS and sepsis patients, whereas a third study on acute bacterial infections found no change in arteriolar caliber [13]

The arteriolar vasoconstriction observed herein may partly explain the absence of nonspecific acute chorioretinal lesions, such as hyporeflective spots or intraretinal hemorrhages, commonly reported in canine and human sepsis [8, 12, 15].

Nevertheless, future studies employing more sensitive diagnostic methods, such as FA and optical coherence tomography, at different time points are necessary to validate these findings [8, 19].

Another possible explanation for discrepancies in arteriolar findings between studies is that human investigations encompassed heterogeneous SIRS etiologies (e.g., surgery, pneumonia, urinary tract infection, soft-tissue infection, and peritonitis), whereas our study focused solely on pyometra [13,14].

### Retinal vascular changes during recovery and study limitations

In humans with sepsis, no differences in retinal vascular parameters were found between survivors and non-survivors within the first 24 h [16]. In the present study, the small number of non-survivors (4/40) precluded statistical comparisons. Previous human studies by Invernizzi *et al*. [12], Fitt *et al*. [13], and Grogan *et al*. [33] have tracked retinal vascular evolution during recovery from SIRS.

One study demonstrated persistent retinal vessel dilation for at least 3 days following surgery-induced SIRS [33]. Another observed normalization of vessel calibers 6 months post-COVID-19 infection [12], while a third noted decreased venular calibers following antibiotic treatment for systemic bacterial infections [13].

All retinal images in this study were obtained at admission; thus, no postoperative retinal follow-up was performed. Future studies should explore longitudinal changes in retinal vessels and potential associations with mortality.

### Correlations between retinal vessel calibers and systemic parameters

In this study, VEGF levels and selected hematological markers showed weak correlations with retinal vessel calibers. Notably, a human study by Liu *et al*. [22] has reported similarly weak correlations, with r-values typically ranging from 0.08 to 0.1.

A meta-analysis in humans indicated that total leukocyte count was more consistently associated with retinal vessel calibers than cytokines or C-reactive protein [22]. Another study reported positive correlations between total leukocyte and neutrophil counts and retinal venular calibers in patients with acute infections [13].

In the present study, no correlation was found between total leukocyte count and retinal vessels. However, segmented neutrophils correlated with arteriolar calibers, and band neutrophils and monocytes correlated with venular calibers.

Neutrophils are critical early responders to infection and contribute to blood vessel wall inflammation through lytic enzyme secretion [34]. Monocyte subsets, including classical inflammatory and non-classical patrolling monocytes, play distinct roles in vascular surveillance and inflammation modulation [35].

One experimental study demonstrated that patrolling monocytes preferentially localize to retinal vessels during early diabetes to mitigate inflammation [35]. Although our findings may reflect similar mechanisms, they should be interpreted cautiously, as the source of inflammation was distant from the eye.

### Role of VEGF, albumin, and IL-4 in retinal microcirculatory changes

Veiga et al. [3] demonstrated increased uterine blood flow and VEGF expression in bitches with pyometra. Similarly, König *et al*. [24] and Gaudette *et al*. [25] reported elevated VEGF levels in septic dogs, however, König *et al*. [24] found no difference between healthy dogs and those with SIRS.

While some P-SIRS patients likely progressed to sepsis, the lack of uterine bacterial cultures represents a limitation. Nevertheless, significantly higher VEGF levels observed in the P-SIRS group suggest the presence of sepsis in some cases.

The observed correlation between VEGF and retinal arteriolar caliber suggests that uterine overexpression of VEGF may contribute to arteriolar vasoconstriction in systemic microcirculation.

Although VEGF is also produced locally in the retina during hypoxia [23], further studies are required to elucidate whether similar retinal processes occur under systemic inflammatory conditions.

Albumin maintains colloid osmotic pressure and prevents interstitial fluid accumulation [1]. Its levels decline during systemic inflammation and infection [1]. In this study, P-SIRS patients exhibited lower albumin concentrations, and a negative correlation was found between albumin levels and venular calibers. Despite these findings, no acute chorioretinal lesions were observed.

Differently, a previous human study by Fitt *et al*. [13] failed to demonstrate a correlation between serum albumin and retinal vessel calibers.

### IL-4 concentrations and clinical implications

IL-4 functions as an anti-inflammatory cytokine and promotes trained immunity [26]. IL-4 concentra-tions were significantly higher in the P-SIRS group, aligning with previous findings in pyometra-affected bitches [5]. In humans, IL-4 and IL-10 levels rise during the recovery phase of SIRS and sepsis, although they typically remain lower than pro-inflammatory cytokines such as IL-6 and TNF-α [26, 36]. Higher IL-4 levels in 10 P-SIRS patients suggest that they were either in an advanced SIRS stage or transitioning toward sepsis. However, further studies using more sensitive assays are needed to confirm the role of IL-4 in pyometra. In this study, IL-4 was detected in only two healthy female dogs and showed no correlation with retinal vessels, suggesting that IL-4 is unlikely to directly regulate the vascular changes observed.

## CONCLUSION

This study demonstrated that bitches diagnosed with pyometra-induced P-SIRS exhibited significant retinal microvascular alterations, characterized by arteriolar constriction and venular dilation, compared with clinically healthy controls. These retinal vascular changes were weakly but significantly correlated with systemic inflammatory markers, including segmented neutrophils, band neutrophils, monocytes, and VEGF concentrations. Notably, higher serum levels of VEGF and IL-4 were detected in P-SIRS patients, suggesting a systemic inflammatory and potentially septic profile. Despite the observed vascular alterations, no chorioretinal lesions, such as hyporeflective spots or hemorrhages, were detected in any of the patients.

The findings suggest that smartphone-based retinal imaging, combined with basic image analysis tools such as ImageJ, provides a feasible, non-invasive method for detecting microvascular alterations associated with systemic inflammatory conditions in veterinary practice. Retinal vascular assessment could potentially serve as an adjunct biomarker for monitoring systemic inflammation or early vascular dysfunction in canine patients with pyometra and related conditions.

This investigation is the first to systematically evaluate retinal vessel calibers in dogs with P-SIRS using smartphone fundus photography. The study applied a rigorous methodology, including blinded assessments, calibration of measurements, intra- and interobserver agreement analysis, and correlation with systemic biomarkers. The focus on a single disease entity (pyometra) enhanced the homogeneity of the sample and minimized confounding factors.

The cross-sectional design limited the ability to assess longitudinal changes in retinal vasculature during disease progression or recovery. The small number of non-survivors precluded survival analysis. Furthermore, the absence of bacterial culture data limited the ability to definitively distinguish between systemic inflammation and sepsis. In addition, slight biases related to calibration methods and absence of refractive media corrections may have affected absolute vessel size measurements.

Future research should incorporate longitudinal follow-up with repeated retinal imaging during treatment and recovery phases to better elucidate dynamic vascular responses. Larger multicentric studies involving a broader range of inflammatory and septic conditions would help validate retinal vessel caliber changes as systemic biomarkers. Advanced imaging modalities such as OCTA and FA should be employed to confirm and extend these findings. Further exploration of cytokine profiles, including dynamic changes in IL-4, VEGF, and other inflammatory mediators, would also be beneficial.

Retinal vascular alterations are detectable in bitches with P-SIRS and show associations with systemic inflammatory markers. Retinal imaging thus offers a promising, minimally invasive tool for adjunctive assessment of systemic inflammation in veterinary patients. Further research is warranted to establish retinal microvascular evaluation as a diagnostic and prognostic modality in veterinary critical care.

## AUTHORS’ CONTRIBUTIONS

FDP: Data curation, investigation, project administration, and drafted the manuscript. APR: Designed the study and reviewed the manuscript. BER: Communicated with owners of dogs and collected samples. HRM: Communicated with owners of dogs and collected samples. NAP: Laboratory analysis. All authors have read and approved the final manuscript. All authors have read and approved the final manuscript.
